# The Socio-Economic Burden of Spinal Muscular Atrophy: A Cost-of-Illness Study in Bulgaria

**DOI:** 10.3390/healthcare13040401

**Published:** 2025-02-13

**Authors:** Elizabet Dzhambazova, Kostadin Kostadinov, Lilia Tsenkova-Toncheva, Fani Galabova, Fares Ezeldin, Georgi Iskrov, Rumen Stefanov

**Affiliations:** 1 Department of Social Medicine and Public Health, Faculty of Public Health, Medical University of Plovdiv, 4002 Plovdiv, Bulgaria; kostadinr.kostadinov@mu-plovdiv.bg (K.K.); lilia.tsenkova@mu-plovdiv.bg (L.T.-T.); georgi.iskrov@mu-plovdiv.bg (G.I.); rumen.stefanov@mu-plovdiv.bg (R.S.); 2 Department of Pediatrics, Medical Faculty, Medical University of Plovdiv, 4002 Plovdiv, Bulgaria; fani.galabova@mu-plovdiv.bg; 3 Pediatrics Clinic, St. George University Hospital, 4002 Plovdiv, Bulgaria; 4 Department of Physical and Rehabilitation Medicine, Medical Faculty, Medical University of Plovdiv, 4002 Plovdiv, Bulgaria; fares.z.ezeldin@gmail.com; 5 Institute for Rare Diseases, 4023 Plovdiv, Bulgaria

**Keywords:** spinal muscular atrophy, costs, burden of disease, cost of illness, quality of life, Bulgaria

## Abstract

**Background/Objectives**: The objective of our study was to quantify the annual costs, from a societal perspective, encompassing direct health care costs, direct non-health care costs, and labor productivity losses associated with spinal muscular atrophy (SMA) patients in Bulgaria and their caregivers. **Methods**: We applied a prevalence-based, bottom-up costing methodology to assess the socio-economic burden of SMA from a societal perspective. We evaluated and summed up all costs for health services (diagnosis, treatment, follow-up, and rehabilitation), educational and social services, and formal and informal care in the community, as well as indirect costs due to the loss of productivity and work capacity of the SMA patients’ caregivers. **Results**: Nine parents of SMA patients provided consent and completed the study’s questionnaire. Two children had SMA type III, and seven had SMA type II. The median annual socio-economic burden per SMA patient was EUR 254,968.80. The high direct costs, primarily driven by drug expenses, and the substantial indirect costs resulting from the loss of productivity among informal caregivers were the primary causes. We found no utilization of social care and educational services. **Conclusions**: We emphasize the need for careful consideration of long-term outcomes, real-world data collection, and performance-based reimbursement. An ideal scenario could achieve these objectives in synergy. A second layer of policy actions and measures must address the unmet needs of SMA patients and their families using a holistic approach. The indirect costs associated with SMA, particularly the productivity loss of informal caregivers, underscore the need for comprehensive support programs.

## 1. Introduction

The socio-economic burden of diseases refers to the overall impact of diseases on society, including both direct and indirect costs. This concept encompasses the financial, social, and psychological effects of illness on individuals, families, and communities [1]. Studies of the socio-economic burden are important, as they provide evidence and support in forming public health policies and strategies [2]. Understanding the socio-economic burden helps governments and health organizations allocate resources more effectively. It highlights which diseases have the most significant economic impact and where interventions can be most beneficial [1]. It helps in designing targeted interventions to promote health equity and ensure that all population groups receive adequate healthcare [3]. By examining the socio-economic burden of diseases, we can better explore the full impact of their broader social and economic consequences, which is essential for holistic health planning.

Spinal muscular atrophy (SMA) refers to a group of rare hereditary diseases that affect motor neurons [4,5,6,7]. The estimated prevalence is 1 in 100,000, while the incidence rate is about 1 in 10,000 live births [8]. Degeneration and loss of lower motor neurons in the spinal cord and brain stem nuclei cause the main symptom of SMA—progressive muscle weakness [7,9]. The main causes of death in SMA patients are primarily related to respiratory complications, including respiratory failure and respiratory infections [10]. The age of onset and severity determine the classification of SMA into four types, which subsequently explain the prognosis in these specific groups of patients [6]. SMA type 1 is the most severe form, with an onset before six months of age. Without treatment, life expectancy is typically less than 2 years. SMA type 4 is the mildest, with an adult onset and a life expectancy close to normal [6,8].

Over the past decade, SMA stakeholders have observed a pronounced surge in interest in this rare condition. Historically, SMA was considered incurable, but recent developments have changed this outlook dramatically. Three new treatments for SMA have been approved for the market: nusinersen, onasemnogene abeparvovec, and risdiplam, authorized in the EU in 2017, 2020, and 2021, respectively [6,9,11,12,13]. These treatments have shown promise in improving motor function and survival rates [7]. This combined approach has resulted in an improved overall prognosis and heightened awareness among policymakers and society.

Those recent developments have affected Bulgaria as well. The estimated incidence of SMA in the country is about 1 in 11,000 live births, resulting in approximately five new cases per year. There were about 160 SMA patients in Bulgaria in 2022, considering all types of SMA, both treated and untreated cases [14]. Regular treatment and follow-up are usually provided in outpatient settings. Four healthcare providers are designated by the National Health Insurance Fund (NHIF) to prescribe and organize medicinal therapies for SMA [15]. Three of those centers are in the country’s capital, Sofia, and one is located in Plovdiv [15]. Patients can choose any of these centers regardless of their place of residence.

Bulgarian SMA patients could benefit from all new therapeutic innovations. Nusinersen and risdiplam were officially approved for reimbursement by public funds in 2019 and 2021, respectively [16]. Onasemnogene abeparvovec, despite not being listed in the Positive Drug List, is available and funded through an individual access scheme [17]. Bulgaria also plans to implement newborn screening (NBS) for SMA. The addition of SMA to the national panel for NBS was approved in 2024 [18], although the actual launch was postponed until 2025.

Despite all of these major health policy changes in the last five years, little is known about the real-world socio-economic burden of SMA in Bulgaria. Kamusheva and Dimitrova published a budget impact analysis in 2020, shortly after the NHIF started reimbursing for nusinersen. This study argued that this was an important, major therapeutic opportunity for SMA patients and supported the public coverage of this medicinal product [19]. A policy analysis of 2021 outlined the framework for coverage of nusinersen [20]. Since then, no additional health economic research on SMA has been conducted in Bulgaria. Understanding the true socio-economic burden of this group of rare conditions is crucial at this point. First, it could help evaluate the past policy actions and their real-world impact on patients and the healthcare system. Second, it could provide important insights and benchmarks for the ongoing public health activities and strengthen their prospective outcomes.

The objective of our study was to quantify the annual costs from a societal perspective, encompassing direct health care costs, direct non-health care costs, and labor productivity losses associated with SMA patients in Bulgaria and their caregivers. Additionally, we aimed to identify the primary factors driving these costs.

## 2. Materials and Methods

### 2.1. Study Design and Data Collection

We conducted a bottom-up, cross-sectional, paper-based survey on the socio-economic burden of SMA in Bulgaria. We designed our research as a single-center study. The questionnaire was distributed and completed during regular patient visits at St. George University Hospital (Plovdiv, Bulgaria). St. George University Hospital is the biggest tertiary healthcare provider in Bulgaria and serves as a regional hub for Southern Bulgaria. The clinic of pediatrics is the only SMA reference center outside of the country’s capital, Sofia [15].

Primary data collection took place between June and November 2024. We chose a 6-month period because the main treatment modality typically requires patients to undergo medicinal treatment and follow-up every 4–6 months [15]. Therefore, we considered this period of time sufficient to cover potentially all SMA patients currently under treatment and follow-up at St. George University Hospital.

### 2.2. Study Participants

Patients under the age of 18 years and with a confirmed diagnosis of SMA type 1, type 2, or type 3 who received regular treatment and follow-up at St. George University Hospital participated in the study. SMA patients were living in the community. We informed the parents of the SMA patients about the rationale and objectives of the study and asked them to indicate their agreement to participate. Respondents who agreed to participate signed an informed consent form. The data collection process did not directly survey SMA patients, as their parents provided all of the required data.

### 2.3. Study Questionnaire

The questionnaire was based on the adapted version of the methodology for assessing the socio-economic burden of rare diseases in Europe that was previously developed within the framework of the international project “Socio-economic Burden and Health-Related Quality of Life in Patients with Rare Diseases in Europe” (BURQOL-RD) [21]. The European Commission co-financed BURQOL-RD, and the BURQOL-RD project consortium included some of this study’s authors.

The questionnaire contained a total of 67 questions that were distributed in five sections: patient’s profile (13 questions), health and social resource utilization (23 questions), educational resource utilization (5 questions), health-related quality of life (6 questions), and caregiver’s profile (20 questions). We followed BURQOL-RD’s bottom-up approach by asking respondents to indicate only the utilization of various goods, services, and resources, but not the actual costs [21,22]. Two health professionals and one patient representative reviewed the draft questionnaire for clarity and consistency.

### 2.4. Costing Methodology

We applied a prevalence-based costing methodology to assess the socio-economic burden of SMA from a societal perspective. This methodological approach considers all existing cases within a specific year and all healthcare resources utilized for diagnosis, treatment, follow-up, and rehabilitation, in addition to other resources employed (formal and informal care) or lost (labor productivity) during that year due to the illness in question [21,22]. The prevalence-based cost-of-illness approach effectively integrates all annual healthcare expenditures, which is appropriate for chronic illnesses like SMA that necessitate prolonged treatment and care [22].

We evaluated and summed up all costs for health services (diagnosis, treatment, follow-up, and rehabilitation), educational and social services, and formal and informal care in the community, as well as indirect costs due to the loss of productivity and work capacity of the SMA patients’ caregivers. The bottom-up costing methodology quantified the total annual costs per SMA case [21,22,23].

Primary data on the use of goods, services, and resources were collected individually from each respondent. We compiled a cost item catalogue of unit costs of all goods, services, and resources. We determined unit costs for goods, services, and resources covered by public funds by consulting relevant government and regulatory databases such as the National Framework Contract and Positive Medicine List, among others (Table 1). We based unit costs for goods, services, and resources not covered by public funds on average market prices. We multiplied unit costs by the reported use of resources to quantify the total annual costs per patient [21,22,23]. The reference year for quantifying all costs was 2024.

Informal care refers to the actions and services performed by non-professional caregivers, typically family members of the SMA patient, who do not get any monetary compensation for their caregiving efforts. Information on informal care was obtained from the survey items based on the time spent assisting the patient with their main daily activities. We measured the duration of informal care as a replacement for paid formal care. This approach, known as the proxy good method, values time as an output [24].

We applied the concept of human capital to assess the indirect costs in terms of loss of productivity and work capacity [22]. We only included this cost category for SMA patients’ caregivers because all SMA patients were children under the age of 18 years. The mean gross earnings per month in September 2024 (the latest publicly available monthly estimate at the time of data collection completion) were used as a reference unit to calculate these costs [25].

We converted and presented all costs per item and the total socio-economic burden of SMA per patient in euros using the current fixed currency exchange rate in Bulgaria (EUR 1 equals BGN 1.95583) [26].

### 2.5. Methodology for Assessing Health-Related Quality of Life

We assessed the health-related quality of life of SMA patients using the self-administered standardized questionnaire EuroQol 5-domain, 3-level version (EQ-5D-3L). EQ-5D-3L is a generic instrument with five domains covering mobility, self-care, daily activities, pain/discomfort, and anxiety/depression [27]. EQ-5D-3L is translated and validated in Bulgarian [27]. There are, however, no EQ-5D-3L tariffs for the Bulgarian population [27,28]. Therefore, we used the time trade-off value set for the UK population [29,30].

### 2.6. Statistical Analysis

We applied descriptive statistics only. We considered statistical inference irrelevant for this study because we anticipated a very small sample of SMA patients and caregivers. Since most of the metric variables had a skewed distribution, we presented them using the median and interquartile range. We presented categorical variables using count and percentage. Descriptive analysis was performed using the Microsoft Excel software package (Microsoft 365 Office Version 2404, Build 17531.20152).

### 2.7. Ethics Committee Approval

The methodology and objectives of this study were reviewed and approved by the ethics committee at the Medical University of Plovdiv (Decision № 5/06.06.2024).

## 3. Results

### 3.1. Patient’s Profile

Nine parents of SMA patients provided consent and completed the study’s questionnaire (Table 2). The age range of the SMA patients was 3–17 years, with teenagers (55.6%) dominating the sample. Boys made up the majority of the participants (55.6%). Two children had SMA type III, and seven had SMA type II. No participants reported SMA type I. The median age at diagnosis was 2 years, with an interquartile range (IQR) of 1.50 to 2.17 years. None of the respondents had a family history of SMA.

A quarter of the patients (22.2%) had comorbidities (Table 2). All but one child needed a caregiver. All respondents (100%) listed a parent as their main caregiver. No one reported using a paid carer. Almost all children with SMA (88.9%) had a disability assessment, providing them with access to publicly funded social services.

### 3.2. Patient Household Profile

All patients’ parents were married (Table 3). The median household size was four members, with an interquartile range (IQR) of three to four members. All fathers held full-time or part-time employment, while less than a quarter (22.2%) of the mothers reported employment. Among the unemployed mothers (n = 7), six (85.7%) cited the need to care for the patient as the reason for their unemployment.

The median gross household income per month was EUR 1278.23, with an IQR of EUR 766.94 to 1789.52 (Table 3). The median SMA-related out-of-pocket payments per month constituted a median of 8% of the monthly household income, with an IQR of 7% to 16%.

The median overall satisfaction with the health system was high (Table 3). On a scale of 1 to 10, it received a rating of 9, with an IQR ranging from 8 to 10.

### 3.3. Health-Related Quality of Life

All patients reported having some or severe problems with mobility (Table 4). Similar findings were observed for the domains of self-care and usual activities. A slight majority of the participants (55.6%) reported no pain or discomfort, and two thirds (66.7%) indicated no anxiety or depression.

The median EQ-5D-3L VAS score was 70 with an IQR of 50–80. The corresponding utility score was 0.516 with an IQR of 0.137–0.710.

### 3.4. Socio-Economic Burden of SMA

The median annual socio-economic burden per SMA patient was EUR 254,968.80 (Table 5). This estimate was to be stable across the sample, with an IQR of EUR 253,036.47–255,917.35 and a range of EUR 251,877.24–261,300.84. The high direct costs, primarily driven by drug expenses, and the substantial indirect costs resulting from the loss of productivity among informal caregivers were the primary causes. We found no utilization of social care and educational services. There were no reports of early retirement among the informal caregivers.

## 4. Discussion

To our best knowledge, this is the first cost-of-illness study about SMA from Southeastern Europe. The median annual socio-economic burden of SMA in Bulgaria is EUR 254,968.80. The primary factors contributing to this outcome were the new disease-modifying drugs, which cost EUR 233,734.76, and the productivity loss of the main informal carer, which cost EUR 11,718.81. A systematic review of SMA burden found comparable, although very variable, results in 2021 [31]. The authors concluded that there was a substantial spread in these estimates, and most research showed some level of bias [31].

We believe that a direct cross-country comparison of SMA burdens between Bulgaria and other countries is not always applicable because health systems operate in different political and economic settings [32]. Patient populations may also differ in terms of SMA type and phenotype [31]. Nonetheless, the role and impact of cost-of-illness studies are important at the local policy level, where this research could inform and support decision-making regarding this rare condition. There is one common feature among all of the published studies on this topic so far—they all highlight the unmet needs of SMA patients and their caregivers [31].

### 4.1. Novel SMA Therapies as the Main Cost-Driving Factor

SMA is a rare genetic disorder characterized by weakness and wasting in skeletal muscles, which can lead to severe physical disabilities and respiratory issues. The socio-economic burden of this condition is multifaceted. Nevertheless, the novel SMA treatments, recently approved for market use, represent the biggest cost-driving factor. This is indeed the case in Bulgaria, as our study reveals that medicinal therapy expenses account for 91.7% of the total median SMA burden. According to a systematic review by Dangouloff et al., the average annual cost for SMA drugs varies [33]. It depends on the price of the specific medication as well as the disease type. This burden typically increases by two times in the case of the most severe type of SMA, known as type 1 [33].

One important factor to consider when discussing the costs for novel medicinal treatments for SMA is the potential discount that payers and industry may negotiate. We reported this cost item in our study using official listed prices. However, the current nearly mandatory use of confidential price discounts significantly influences these estimates [34]. In Bulgaria, for example, the mandatory price discount for reimbursement agreements is at least 10% by law [17]. This requirement, of course, is limiting the transparency of drug costs and subsequently the cross-country comparison, as different jurisdictions have different levels of leverage in these negotiations [17,34,35].

In Bulgaria, the novel disease-modifying therapies for SMA, namely nusinersen, onasemnogene abeparvovec, and risdiplam, are reimbursed under different access schemes. However, their overall budgetary impact is tremendous. Official reimbursement of nusinersen and risdiplam by the NHIF has resulted in total costs of EUR 76,746,498.13 to the end of August 2024 [36]. Additionally, onasemnogene abeparvovec, although not officially included in the Positive Drug List, is eligible for coverage under an individual access scheme. Four SMA patients were treated with this medicinal product in 2021 and 2022, for a total sum of EUR 11,402,550.16 [17].

Novel SMA medicinal treatments offer new therapeutic opportunities for SMA patients, addressing significant unmet medical needs. However, these medicinal products come with high costs, raising questions about their cost-effectiveness, accessibility, and overall budget impact, especially in a healthcare system with limited resources [33,37,38]. In this context, it is crucial to monitor the long-term outcomes of these treatments and link them to the coverage decisions and payments [39]. International data look promising to this point [40,41]. Bulgaria typically collects data on health outcomes every 6 months, but to our knowledge, no publicly available information exists about these results and their implications. Therefore, we would recommend making these data available and accessible for public use and linking them to actual reimbursement negotiations. Public spending should reflect the actual economic value of these novel therapies, translating real-world clinical value into coverage decisions [33,38,39].

### 4.2. Opportunities for SMA Newborn Screening

Our findings suggest that the upfront costs of SMA novel treatments are high. Nevertheless, the long-term benefits in terms of health outcomes and cost savings could potentially justify the investment, especially when treatment is initiated early [42]. This is why the promptness and precision of SMA diagnosis have become more crucial than ever, as the therapeutic efficacy may be significantly diminished if the treatment cannot be started at the appropriate time due to a delayed or incorrect diagnosis [43].

NBS prevents this diagnostic delay and allows early treatment for both pre-symptomatic and symptomatic SMA patients within 3–4 weeks post-birth [43]. The early onset of treatment greatly enhances the therapeutic outcomes. Over 90% of those early-treated children with three or more SMN2 copies reach normal developmental milestones, compared to only 34% without early intervention. Those treated after symptoms appear rarely achieve independent walking unless this was attained before the disease onset [44]. NBS for SMA could significantly improve motor function outcomes and reduce the need for supportive interventions when compared to clinical diagnosis by referral [45].

Screening for SMA is among the recent additions to the panels of NBS programs worldwide [46]. The rationale behind NBS for SMA is, of course, a direct consequence of the availability of reimbursed novel therapies in the majority of European countries and the United States [47]. By the end of 2020, nine countries across the world had initiated NBS for SMA, with more nations planning to implement this public health measure [47]. Studies evaluating SMA NBS in Germany and Belgium conclude that, considering the available therapies for SMA and the importance of treatment prior to symptom onset, NBS programs should incorporate SMA testing for all infants [48,49].

SMA was officially approved for inclusion in the Bulgarian national NBS program in 2024, and these activities are expected to start in 2025 [18]. We believe these two activities—NBS for SMA and the reimbursement of novel therapies for this condition—could and should be implemented in synergy. Creating a national registry of SMA patients in Bulgaria could help to collect and analyze real-world data about the effects of SMA therapies [50,51,52,53,54]. This could subsequently set the stage for genuine outcome-based reimbursement and increased value for money.

### 4.3. The Need for Comprehensive Support Programs for SMA Families and Caregivers

We found the indirect costs due to the productivity loss of the main informal caregiver to be the second most important cost-driving factor for the overall socio-economic burden of SMA. The median annual amount of social allowance (EUR 5706.02) was only about half of the median annual productivity loss of the main carer (EUR 11,718.81). This discrepancy creates a critical gap that puts substantial financial pressure on the families of SMA patients in Bulgaria. Parents are practically forced to limit all SMA-related out-of-pocket payments, thereby restricting the use of psychological care and educational support for children with SMA. Only one respondent indicated the use of such paid services in our study.

Strengthening the psychological and educational support for SMA patients and their families is one key area that our study suggests. Various studies have confirmed the enormous burden on SMA patients’ caregivers [55,56,57,58]. While the new medicinal treatments for SMA represent significant progress, caregiver overburden due to everyday care tasks remains high [56,57]. Family members and caregivers of SMA patients report experiencing depression, anxiety, stress, a worsened quality of life, and limited or no ability to work [57]. Our study showed that one of the parents had to practically stop working in order to take care of their child. Therefore, there is an urgent need for family-centered help services to address the needs of these patients and their caregivers [58].

Public respite care services in Bulgaria are limited in terms of scope and availability. In the context of chronic conditions that require substantial amounts of informal care, expanding access to respite care services should be a major policy priority. Other studies have confirmed this important necessity [59]. A specific aspect of the care burden for SMA involves the need for respite care to alleviate exhaustion, along with the provision of physiotherapy services. An SMA patient’s caregiver is prone to injuries due to the frequent lifts and transfers of the patient [59].

Finally, the restricted use of psychological care, as indicated by our results, is concerning. This outcome is likely due to financial constraints. Parents, serving as primary informal caregivers, experience physical and psychological strain [58,59]. Comprehensive support programs should include facilitated access to mental health services for both children with SMA and their caregivers. This is very important because psychological care could help to address the emotional and psychological challenges that these people face on a daily basis [56].

### 4.4. Health-Related Quality of Life in SMA Patients

The therapeutic advances in SMA have put additional focus on the health-related quality of life in these patients and their caregivers [60,61]. Even though all patients from our study reported certain health problems and restrictions in their everyday life, we noticed relatively high levels of both EQ-5D-3L VAS and utility scores. Wohnrade et al. used the EQ-5D-5L in similar research. SMA patients were defined as having a low or high health-related quality of life if their utility scores were less than 0.259 or more than 0.679 [62]. In our case, we found a median utility value of 0.516 with an IQR of 0.137–0.710. According to Wohnrade’s cutoff values, we identified four and three participants with a high and low health-related quality of life, respectively. Nonetheless, we saw very high overall satisfaction with the health system. Our study’s participants indicated a median satisfaction rating of 9 (on a 1–10 scale) with an IQR ranging from 8 to 10.

The quality of care, including the availability and accessibility of new SMA medicinal treatments, influences health-related quality of life and patient satisfaction [63]. Therefore, it is important to consider the therapeutic context and the treatments available when comparing patient-reported outcomes from different countries and jurisdictions. For example, Lopez et al. reported relatively lower EQ-5D-3L results in SMA patients from Spain [64]. However, the original collection of these data took place in 2015, prior to the introduction of SMA disease-modifying treatments. More recent studies from Germany and Sweden found improved health outcomes, potentially implying the impact of new SMA therapies [62,65]. Still, there is a substantial spread in the quality-of-life estimates in SMA patients, underlying the need for more research [65,66]. Besides the type of SMA, diagnostic delay, and onset of treatment, other authors have also mentioned the proximity to healthcare services as a factor for improved outcomes in SMA patients [67].

Last but not least, we must also consider the type of tool applied to collect and measure health-related quality of life in SMA when comparing those data. SMA-specific instruments may be more appropriate to reflect the disease course, as well as the impact of new medicinal therapies [63]. A review identified a total of 36 tools used to collect health-related quality of life data in SMA, with 6 of them being specifically designed for this rare condition [61]. It is now generally recommended that patient-reported outcome measures be used and that this kind of information be collected regularly when SMA patients are being followed up [68,69].

### 4.5. Limitations

Our research findings are subject to several limitations. First, the sample size is very small. This restricts the practical application of statistical inference and generalizability. Nevertheless, our survey was designed as a single-center study, collecting data for a very limited time period. We had a perfect participation rate as, to our best knowledge, no parent of a child with SMA declined to take part in this study. Therefore, we included all patients treated at the only reference center for this rare disease outside of the country’s capital. Moreover, some of the previous SMA burden-of-disease studies have used comparable samples in terms of size, considering the country’s total population and the data collection period [31,33].

Second, the main inclusion criterion for our study was the diagnosis of SMA type 1, type 2, or type 3. However, our research included only SMA type 2 and type 3 patients. There were no SMA type 1 cases, which represent the most severe subset of this rare condition. There is a lack of publicly available data regarding the epidemiology of SMA in Bulgaria. Therefore, we cannot assess the prevalence of different SMA types in the country. Of course, we could suggest that SMA type 1 patients and their caregivers would very likely experience a bigger burden and worse health outcomes. We should test this hypothesis in the future by conducting a larger study, potentially including the other three SMA reference centers based in Sofia.

Third, all nine patients from this study were treated with nusinersen. This is one of the new disease-modifying therapies for SMA that are currently available in Bulgaria. Public funds also cover onasemnogene abeparvovec and risdiplam. Collecting similar information about SMA patients currently treated with these two other options is crucial to identify potential similarities and differences. Such real-world data would actually be very helpful in light of the outcome-based reimbursement paradigm.

Fourth, our study used a generic instrument (EQ-5D-3L) to assess the health-related quality of life of SMA patients. This is a broad tool to explore health-related quality of life and to compare outcomes among different interventions and conditions. Nevertheless, we recommend combining the application of this tool with SMA-specific instruments in future studies to gain a better understanding of this rare disease’s impact on patients [61,63]. Additionally, we did not aim to evaluate the health-related quality of life of SMA patients’ caregivers. This decision was based on the idea to keep the questionnaire compact and ensure better compliance among respondents. Future research must also address this knowledge gap.

## 5. Conclusions

We conducted the very first cost-of-illness study about SMA in Southeastern Europe. The socio-economic burden of SMA in Bulgaria is substantial, driven primarily by the high costs of novel disease-modifying therapies. We emphasize the need for careful consideration of long-term outcomes, real-world data collection, and performance-based reimbursement. An ideal scenario could achieve these objectives in synergy.

A second layer of policy actions and measures must address the unmet needs of SMA patients and their families using a holistic approach. The indirect costs associated with SMA, particularly the productivity loss of informal caregivers, underscore the need for comprehensive support programs. These programs should include financial assistance, psychological support, and respite care services to alleviate the burden on SMA families and improve their quality of life.

While our study has its limitations, it lays the groundwork for more extensive research. We recommend including a broader range of SMA types, phenotypes, and therapies, as well as involving multiple reference centers. Such data will be essential for understanding the full spectrum of treatment outcomes and informing outcome-based reimbursement decisions.

## Figures and Tables

**Table 1:** Main sources for unit costs of reimbursed healthcare goods, services, and resources.

Cost Item	Source	Link
Drugs	National Council on Prices and Reimbursement of Medicinal Products. Positive Drug List	https://portal.ncpr.bg/registers/pages/register/list-medicament.xhtml (accessed on 9 December 2024)
Medical services (tests, visits, hospitalizations)	National Health Insurance Fund. National Framework Contract 2023–2025	https://www.nhif.bg/bg/nrd/2023-2025/medical (accessed on 9 December 2024)
Dental services	National Health Insurance Fund. National Framework Contract 2023–2025	https://www.nhif.bg/bg/nrd/2023-2025/dental (accessed on 9 December 2024)
Dietary foods	National Health Insurance Fund	https://www.nhif.bg/bg/medicine_food/diet (accessed on 9 December 2024)
Medical devices	National Health Insurance Fund	https://www.nhif.bg/bg/medicine_food/medical https://www.nhif.bg/bg/medicine_food/nonmedical (accessed on 9 December 2024)
Medical materials	National Health Insurance Fund	https://www.nhif.bg/bg/facilities/main (accessed on 9 December 2024)

**Table 2:** Patient profile.

Variable	n	%
Number of respondents	9	-
Median age in years (IQR)	14 (10–17)	-
Age range (min–max)	3–17	-
Age group		
Toddler (1 to 3 years)	1	11.1%
Child (4 to 12 years)	3	33.3%
Teenager (13 to 18 years)	5	55.6%
Sex (male)	5	55.6%
Diagnosis		
SMA type II	7	77.8%
SMA type III	2	22.2%
Median age at diagnosis in years (IQR)	2 (1.50–2.17)	-
Age at diagnosis range (min–max)	(1–17)	-
Family history of SMA (yes)	0	-
Comorbidities (yes)	2	22.2%
Need of carer (yes)	8	88.9%
Parent as a main carer (yes)	8	100.0%
Use of paid carer (yes)	0	-
Disability assessment (yes)	8	88.9%

**Table 3:** Patient household profile.

Variable	n	%
Number of respondents	9	-
Marital status of patient’s parents (married)	9	100.0%
Median household size (IQR)	4 (3–4)	-
Mother’s employment status (full- or part-time employment)	2	22.2%
Reason for unemployment (need to care for the patient)	6	85.7%
Father’s employment status (full- or part-time employment)	9	100.0%
Median gross household income per month in euros (IQR)	1278.23 (766.94–1789.52)	-
Median SMA-related out-of-pocket payments per month in euros (IQR)	102.26 (51.13–286.32)	-
Median share of SMA-related out-of-pocket payments from the monthly income (IQR)	8% (7–16%)	-
Median overall satisfaction with the health system on a 1–10 scale (IQR)	9 (8–10)	-

**Table 4:** Patients’ health-related quality of life (EQ-5D-3L descriptive system).

Domain	No Problems		Some/Severe Problems	
	n	%	n	%
Mobility	0	-	9	100.0%
Self-care	1	11.1%	8	88.9%
Usual activities	1	11.1%	8	88.9%
Pain/discomfort	5	55.6%	4	44.4%
Anxiety/depression	6	66.7%	3	33.3%

**Table 5:** Socio-economic burden of SMA in Bulgaria (in euros).

Cost Item	Median	Q1–Q3	Min–Max
Drugs	233,734.76	233,734.76–233,734.76	233,734.76–233,858.70
Tests	74.93	65.21–83.68	41.91–106.98
Visits	2036.99	1316.37–2150.14	582.87–8892.34
Hospitalizations	1049.32	1049.32–1049.32	1049.32–2098.65
Materials	0.00	0.00–715.81	0.00–968.39
Transport	245.42	122.71–613.55	0.00–3067.75
Social services	-	-	-
Social allowances	5706.02	2760.98–5706.02	0.00–5706.02
Educational services	-	-	-
Professional carer	-	-	-
Total direct costs	242,664.05	240,455.26–243,026.66	237,597.29–251,730.48
Main informal carer’s loss of productivity	11,718.81	11,718.81–11,718.81	5859.40–11,718.81
Main informal carer’s early retirement	-	-	-
Other informal carers’ loss of productivity	1171.88	585.94–2561.14	585.94–4687.52
Other informal carers’ early retirement	-	-	-
Total indirect costs	12,304.75	12,304.75–12,890.69	9570.36–16,406.33
Total costs per year	254,968.80	253,036.47–255,917.35	251,877.24–261,300.84

## Data Availability

The raw data supporting the conclusions of this article will be made available by the authors on request.
